# Scientific Advances in Neural Regeneration After Spinal Cord Injury

**DOI:** 10.7759/cureus.78630

**Published:** 2025-02-06

**Authors:** Mohammed Gartit, Mohammed Noumairi, Abdelilah Rhoul, Houssam Mahla, Younes El Anbari, Ahmed Amine EL Oumri

**Affiliations:** 1 Physical Medicine and Rehabilitation, Faculty of Medicine and Pharmacy, Mohammed First University, Oujda, MAR; 2 Physical Medicine and Rehabilitation, Mohammed VI University Hospital, Oujda, MAR; 3 Faculty of Medicine, Mohammed First University, Oujda, MAR

**Keywords:** and tissue engineering, cell therapy, extracorporeal shock wave therapy, neuromodulation, neuroprotection, neuroregeneration, rehabilitation, spinal cord injury

## Abstract

Spinal cord injury (SCI) is a devastating condition that results in loss of motor and sensory function, morbidity, and severe dependence. Neural regeneration, which refers to the regrowth or repair of nerve tissue or cells, holds promise as a therapeutic approach for SCI. This narrative review explores the current state of neural regeneration for SCI treatment, including endogenous neuroprotection, neuroplasticity, neuroremediation, and cell-based therapies. The review is based on a search of literature from the past 20 years, conducted via databases including PubMed and Google Scholar. While many of the studies show promising results, the majority are preclinical, highlighting the need for randomized clinical trials (RCTs) to evaluate the effectiveness of these therapies in humans. Among the different therapeutic approaches, cell-based therapies, particularly the use of neural stem cells, have shown the most promising results in promoting neural regeneration and functional recovery in animal models and human trials. Therefore, neural stem cell transplantation is considered the most useful treatment method for SCI. However, further research is needed to optimize the transplantation procedure, improve cell survival, and enhance functional outcomes in patients with SCI.

## Introduction and background

Traumatic spinal cord injury (TSCI) represents a critical global health challenge [1,2], leading to high mortality and morbidity rates and burdening healthcare systems on a worldwide scale [1,3,4]. It is one of the leading causes of disability, particularly affecting younger individuals and significantly reducing years lived with a high quality of life [5,6]. Despite significant progress in research efforts exploring various therapeutic modalities, only a limited number of these approaches have been tested in clinical trials. This highlights an urgent need for further investigation into effective treatments for TSCI to improve patient outcomes and alleviate the substantial societal burden associated with this condition.

In this narrative review, we aim to comprehensively explore the current landscape of therapeutic and rehabilitation strategies designed to stimulate neural regeneration in patients with TSCI. Drawing from extensive literature searches across reputable databases such as PubMed, Scopus, and Google Scholar, we critically evaluate the efficacy and potential of these interventions. However, it is essential to emphasize the necessity for rigorous randomized clinical trials (RCTs) to validate the promising findings observed in preclinical studies and to provide robust evidence of therapeutic effectiveness in human subjects. Our review underscores the critical importance of addressing TSCI as a global health priority and emphasizes the need for innovative therapeutic approaches. By shedding light on the current state of research and clinical practice, our findings serve as a valuable resource for clinicians, researchers, and policymakers alike. With this knowledge, we can collectively work towards advancing therapeutic strategies for TSCI, ultimately aiming to enhance patient outcomes, increase quality of life, and reduce the burden of disability on a global scale.

**Figure 1:**
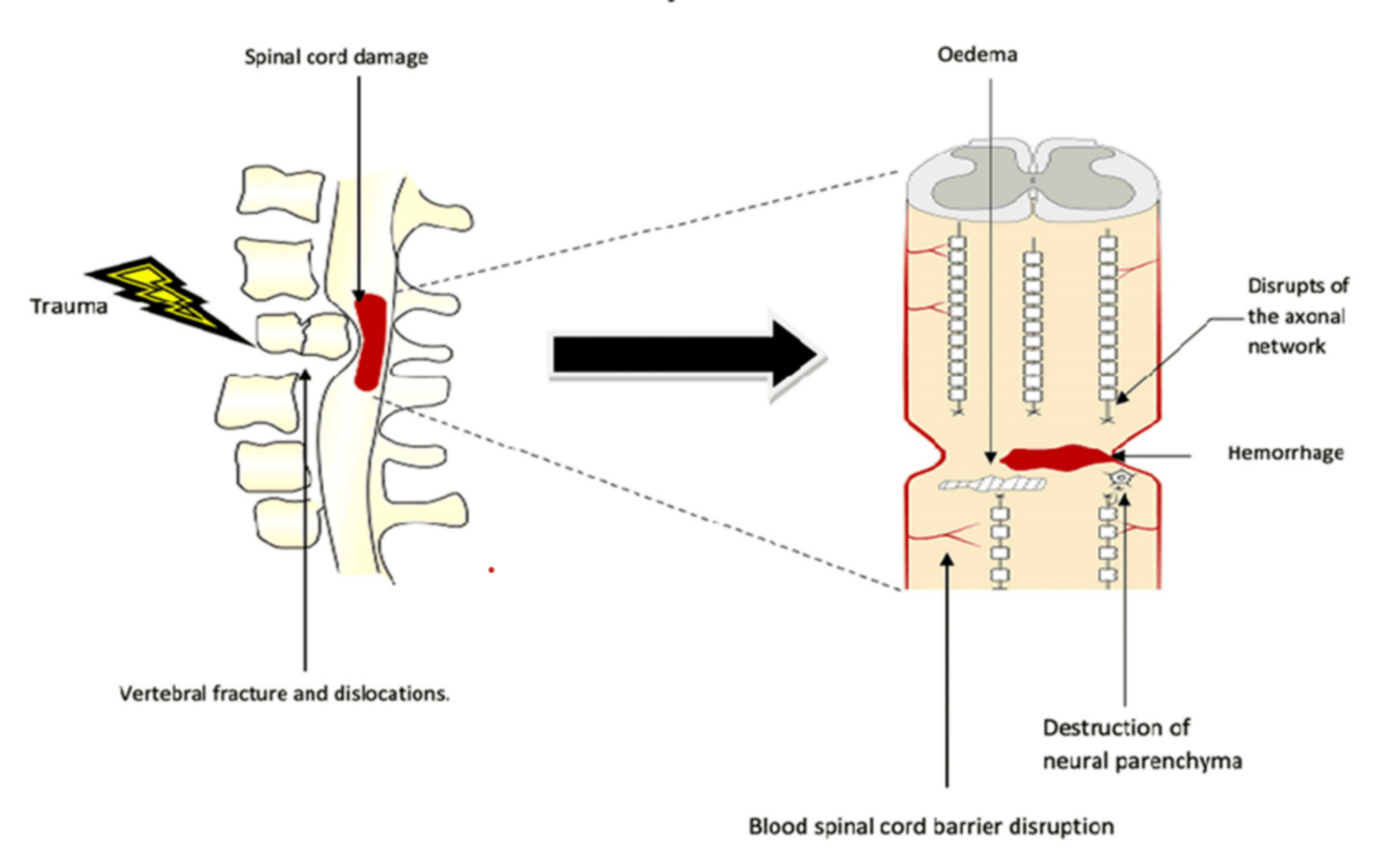
Primary lesion This image illustrates the immediate effects of trauma, including vertebral fractures, dislocations, and spinal cord damage leading to axonal disruption, edema, hemorrhage, and destruction of neural parenchyma Image created by the author

Regarding the pathophysiology of TSCI, it is essential to understand that it involves two forms of damage: the initial or primary injury and subsequent or secondary injury. The primary injury occurs immediately following the traumatic event, resulting in acute damage to the spinal cord, including vertebral fractures, dislocations, and tissue destruction [7,8]. This phase disrupts the neuronal architecture, damages axonal connections, induces hemorrhage, and alters the integrity of the glial membrane (Figure 1).

**Figure 2:**
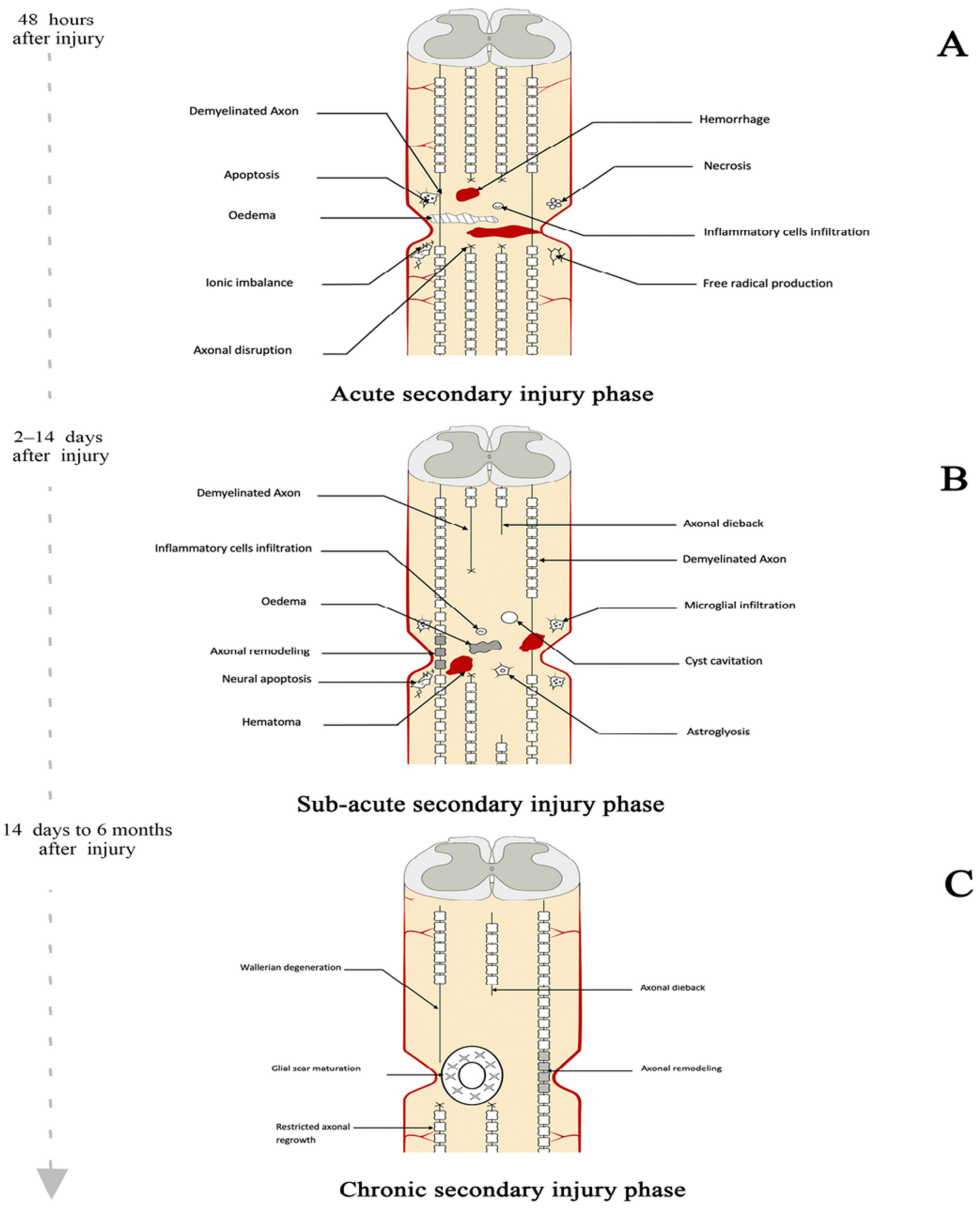
Secondary lesion progression The three phases of secondary spinal cord injury are depicted: (A) Acute phase (48 hours post-injury), showing axonal disruption and inflammatory responses; (B) Sub-acute phase (2–14 days post-injury), featuring apoptosis and axonal remodeling; and (C) Chronic phase (14 days to six months post-injury), marked by Wallerian degeneration and glial scar formation Image created by the author

Following the primary injury, the secondary injury cascade unfolds in a series of acute, subacute, and chronic phases, each characterized by distinct pathological processes (Figure 2). The acute phase is marked by vascular injury, ion imbalances, excitotoxicity, oxidative stress, inflammation, and edema formation. Subsequent phases involve neuronal apoptosis, axonal demyelination, Wallerian degeneration, axonal remodeling, and the formation of a glial scar [9]. In the chronic phase, the spinal cord undergoes further degeneration, leading to the formation of cystic cavities, axonal dieback, and maturation of the glial scar. These processes collectively contribute to the chronicity of SCI and the persistence of neurological deficits [10]. Understanding these pathophysiological mechanisms is crucial for developing targeted therapeutic interventions aimed at mitigating secondary injury processes and promoting neural regeneration in TSCI patients.

## Review

Current treatments

Pharmacological Approach

Methylprednisolone (MP) aids in reducing inflammation and swelling in the spinal cord, which is a significant factor contributing to secondary injury after the initial trauma. Through its anti-inflammatory effects, MP may also protect nerve cells from further damage and promote nerve regeneration. This medication was once considered the gold standard for SCI treatment based on the National Acute Spinal Cord Injury Study (NASCIS) II and III trials [11,12]. However, subsequent research and reanalysis have shown little to no neurological improvement with MP treatment and an increased risk of adverse effects, including death [13]. It is crucial to acknowledge that the administration of MP after SCI carries risks, and the decision to use this medication should be made by a medical professional by taking into account the specific circumstances of each patient.

Overall, while these medications show potential for SCI treatment, further research, including larger trials, is necessary to establish their efficacy and safety conclusively.

Neuromodulation after spinal cord injury

Spinal Cord Stimulation

Preclinical studies have demonstrated that electrical stimulation of the spinal cord can restore impaired neurological functions when applied to the spinal segments containing the neurons responsible for these functions [14-17]. Case studies in humans with SCI have built on this principle, reporting immediate improvements in various neurological functions, including standing and walking, in response to electrical spinal cord stimulation [14,18-20].

Transcutaneous spinal cord stimulation (tcSCS): tcSCS is a noninvasive technique that involves placing electrodes on the skin above the spine. This method has been shown to enhance the excitability of local spinal networks through dorsal root afferents, with additional signal amplification along the entire spinal cord [21-23]. Case studies have demonstrated that tcSCS improves upper extremity grip strength and, when combined with training at different spinal levels, promotes voluntary movements in individuals with chronic SCI [24,25].

Epidural spinal cord stimulation (ESCS): ESCS has been utilized for various purposes, including chronic pain management and functional recovery after SCI. Preclinical research by Gerasimenko et al. has highlighted ESCS's ability to initiate hindlimb stepping in rats with complete SCI [26]. Clinically, ESCS is the most studied neuromodulatory technique in the context of neurotrauma [27,28,19-29]. Gill et al. [27] reported the first case of a chronic, clinically motor-complete SCI patient regaining independent stepping ability with task-specific training supported by ESCS three years post-injury. While the patient achieved bilateral stepping on a treadmill, overground stepping required assistance with a walker and trainer. Similarly, Angeli et al. [28] examined four patients who showed improved locomotor function following intensive treadmill training with weight support combined with ESCS, even after failing to improve with training alone.

Both tcSCS and ESCS have demonstrated significant potential in restoring neurological functions in individuals with SCI. tcSCS provides a noninvasive means to enhance voluntary movements and upper limb functions, while ESCS has shown notable success in supporting locomotor recovery, as evidenced by preclinical and clinical studies. These advancements underscore the transformative potential of spinal cord stimulation in SCI rehabilitation.

Brain Stimulation

Transcranial direct current stimulation (tDCS): tDCS is a noninvasive brain stimulation method that involves applying electrodes to the scalp to deliver a low-intensity direct current. Often combined with motor practice, tDCS enhances brain plasticity. Numerous studies have highlighted its beneficial effects on motor function in both upper and lower extremities for individuals with SCI [30-32]. Research has shown that even a low current density of 2 mA can induce measurable effects in tetraplegic participants, with the intensity of the current influencing functional outcomes [33]. However, the current literature on the efficacy of tDCS for functional motor recovery following SCI is unclear and controversial. While many studies report positive outcomes, others have shown no significant effects [34,35]. Despite its potential, further research is essential to clarify the role of tDCS in SCI rehabilitation and establish standardized protocols for its effective use.

Transcranial magnetic stimulation (TMS): TMS is another noninvasive method that stimulates cortical neurons by sending magnetic pulses through the scalp and skull. Furthermore, TMS seems to exhibit anti-inflammatory properties. It enhances the anti-inflammatory polarization of microglia, helping to reduce neuroinflammation and apoptosis, while also supporting nerve tissue regeneration to some extent [36]. In addition, repetitive transcranial magnetic stimulation (rTMS) improves lower extremity motor function. Studies investigating lower limb function in patients with SCI have demonstrated that TMS, whether used alone or in combination with routine rehabilitation training, can enhance lower limb muscle strength and gait function [37,38].

Rehabilitation

Rehabilitation is an often overlooked yet vital noninvasive approach for enhancing endogenous trophic factor release and promoting long-term cell survival. Despite its potential, it remains underrepresented in preclinical studies. Physical rehabilitation, with or without the addition of electroceutical interventions, plays a crucial role in the treatment of patients with SCI. Functional improvements are observed even with rehabilitation strategies such as forced treadmill training, free swimming, or task-specific assessments like forelimb testing [39,40].

The survival rate of transplanted neural stem cells (NSCs) increases more than fivefold with treadmill-based locomotor training, which enhances Insulin-like growth factor-1 (IGF-1) signaling while also improving cardiorespiratory and musculoskeletal functions [41]. These findings underscore the significance of interdisciplinary and multimodal approaches to SCI care.

The benefits of exercise for individuals with SCI have been thoroughly documented in prior systematic reviews and meta-analyses [42-44]. Collectively, the research supports that exercise interventions lead to improved fitness outcomes for individuals with chronic SCI [45]. While many studies have focused on enhanced cardiorespiratory fitness from aerobic exercise, recent reviews emphasize the additional benefits of resistance training, including gains in muscle strength, endurance, and power for individuals with SCI [46].

Advancements in rehabilitation techniques have introduced innovative and targeted approaches to enhance motor recovery and optimize patient outcomes. Strategies such as high-intensity, high-volume exercise training, functional electrical stimulation (FES), exoskeleton-assisted rehabilitation, and virtual reality (VR) have demonstrated significant potential in improving strength, mobility, and overall functionality. Beyond motor recovery, these methods also address the broader health challenges associated with SCI, including cardiovascular, musculoskeletal, and respiratory complications.

The following sections will delve into various rehabilitation modalities, outlining their mechanisms, effectiveness, and potential benefits for patients with SCI.

Exercise Training

Emerging rehabilitation strategies for SCI emphasize high-intensity, high-volume, and repetitive exercises, which have demonstrated significant clinical benefits in both complete and incomplete SCI cases [47]. Comparative studies on high-intensity interval training (HIIT) and moderate-intensity continuous training (MICT) reveal comparable improvements in aerobic capacity, though neither approach has been proven to be superior [48-50]. Additional findings report benefits such as improved glycemic control, better blood lipid profiles, increased fat-free mass in the lower extremities, enhanced muscle strength, and improved vascular health [49,51]. Further evidence from nondisabled populations highlights system-wide improvements resulting from MICT and HIIT, with additional support for HIIT’s superior benefits in certain domains [52, 53].

Functional Electrical Stimulation

FES, a subset of neuromuscular electrical stimulation (NMES), involves the use of electrical signals to activate paralyzed nerves or muscles, inducing contractions that enable functional movements [54]. Historically, FES has been used in neurorehabilitation for rowing and cycling [55]. FES is extensively applied in SCI rehabilitation, primarily to restore functionality in the extremities. Research has demonstrated its ability to improve muscle power output and endurance [56]. Among its well-documented benefits, FES is noted for increasing muscle size, strength, and composition, which enhances oxidative capacity [57] and reduces fatigue [58].

Numerous studies have validated the efficacy of FES in enhancing upper extremity function following SCI [59,60]. In lower extremity rehabilitation, FES has been shown to improve gait parameters, including foot acceleration, swing power, and ground impact force, resulting in faster walking speeds and more efficient muscle strength for gait [61-63]. Furthermore, emerging research indicates that electrical stimulation can improve the success rate of spinal fusion [64].

FES continues to be a promising tool in the field of neurorehabilitation, with its applications expanding beyond traditional uses to include innovative approaches such as enhancing spinal fusion outcomes. Future research should focus on optimizing FES protocols to further improve functional outcomes and exploring its potential in combination with other advanced rehabilitation technologies, paving the way for even greater strides in patient recovery.

The Application of Exoskeletons for Individuals With Spinal Cord Injuries

Exoskeletons offer a cutting-edge approach to rehabilitation and daily living for individuals with SCI. These devices address critical challenges such as muscle atrophy and the substantial energy demands associated with post-SCI tasks, which often hinder effective rehabilitation efforts [65]. Both passive and powered exoskeletons enhance functional efficiency by supporting weakened muscles and reducing energy expenditure for patients and therapists alike [66]. For example, the Hybrid Assistive Limb (HAL) exoskeleton leverages electromyographic signals to facilitate movement in individuals with incomplete SCI, demonstrating promising outcomes in improving activities of daily living (ADLs) [67,68]. Although exoskeletons are not yet ready to be full replacements for walking, ongoing clinical trials indicate their potential to improve cardiopulmonary function, muscle physiology, and walking performance in individuals with chronic SCI [69-71]. Robotic exoskeleton-based gait training has been shown to benefit cardiovascular health [72], promote bone health [73], and provide psychological advantages [70].

This study builds on previous research supporting the safety and feasibility of intensive robotic exoskeleton training for individuals with SCI [74-76]. A randomized controlled trial aimed to determine whether robotic exoskeleton gait training could significantly improve independent walking speed in individuals with chronic incomplete spinal cord injury (iSCI). Results confirmed the safety and feasibility of exoskeleton training in outpatient settings; however, the average increase in independent walking speed was not statistically significant [77].

Virtual Reality Training

VR is a rapidly evolving technology with promising applications in the rehabilitation of various neurological disorders [78]. Previous studies have reported positive effects of VR training (VRT) on motor recovery in individuals with stroke [79,80] and traumatic brain injuries [81]. While VR is widely employed in the rehabilitation of SCI patients [82], its effectiveness remains a subject of debate. A meta-analysis reported that VR can significantly improve motor function in SCI patients [83], whereas another found no significant impact of VRT on upper limb motor function in this population [84].

A randomized controlled trial [85] evaluated the impact of adding VRT to conventional therapy on sitting balance in individuals with SCI. Participants were divided into two groups: one received conventional therapy alone, while the other received a combination of VRT and conventional therapy. Both groups underwent equal durations of therapy. Results showed that the VR group significantly outperformed the conventional group in the Modified Functional Reach Test (MFRT), but no differences were observed between the groups in the T-shirt test.

Treatments undergoing human trials

Pharmacological Approach

Riluzole, a pharmacological compound that acts as an inhibitor of glutamatergic processes, has exhibited considerable potential in enhancing motor scores among patients suffering from SCI during the initial phase I clinical trial [86]. Nonetheless, it should be noted that the neurological advancements observed in this trial are presently lacking substantial scientific evidence [87]. Subsequently, a more comprehensive phase II/III trial was conducted to assess the effectiveness of riluzole when used as a treatment for acute SCIs. During this trial, patients diagnosed with cervical SCI were randomly divided into two groups: one receiving riluzole and the other administered a placebo, both treatments initiated within 12 hours of the injury. A total of 193 patients were enrolled for this study. Notably, patients who received riluzole showcased considerable improvements in their Upper Extremity Motor scores, particularly those classified as American Spinal Injury Association (ASIA) Impairment Scale (AIS) C. Although the initial assessment fell short of meeting the predetermined criteria for efficacy, further analyses revealed significant functional recovery gains across all subgroups of cervical SCI patients who were treated with riluzole. A phase 2 adaptive, multicenter, placebo-controlled, randomized, double-blind trial, to assess whether riluzole is a safe and biologically effective means of managing spasticity in adult patients with traumatic chronic SCI is already in progress [88].

These compelling findings undeniably warrant additional investigation and attention from guideline development groups, especially considering the lack of accepted neuroprotective treatments for SCI at present [89]. Minocycline, known for its neuroprotective properties, demonstrated a 6-point improvement in motor scores compared to placebo in a phase II trial [90, 91]. A phase III study of minocycline in acute spinal cord injury to assess the efficacy of IV minocycline in improving neurological and functional outcomes after acute non-penetrating traumatic SCI is still ongoing (clinical trials.gov ID NCT01828203). However, larger multicenter trials are needed to validate these findings.

Neuromodulation after spinal cord injury

Digital Bridge From Brain to Spinal Cord

To establish the digital bridge between the brain and spinal cord, Lorach et al. [92] combined two fully implanted systems that facilitate the wireless and real-time recording of cortical activity while also enabling stimulation of the lumbosacral spinal cord. In their study, a 38-year-old male participant with chronic tetraplegia, resulting from a C5/C6 spinal cord injury sustained during a biking accident, utilized a brain-spine interface (BSI) to regain control over his legs. Although he had previously achieved some mobility through a five-month neurorehabilitation program in the Stimulating Movement With Implanted Electrodes (STIMO) clinical trial, he experienced a plateau in his progress. The BSI allowed him to stand, walk, and navigate complex terrains. This system exhibited reliable performance for over a year, including during independent home use, and contributed to his neurological recovery, enabling him to walk with crutches even when the BSI was not activated.

Deep Brain Stimulation

Deep brain stimulation (DBS) is a sophisticated neuromodulation technique that involves implanting electrodes in specific brain regions to deliver electrical impulses, thereby regulating neural activity. Extensively researched in both scientific and medical fields, DBS is primarily utilized for treating movement disorders such as Parkinson’s disease, essential tremor, and dystonia. However, its scope is expanding to include applications in psychiatric conditions, cognitive enhancement, and neurorehabilitation.

Preclinical research

Studies conducted in preclinical settings have shown that stimulating the mesencephalic locomotor region (MLR) leads to significant improvements in gait impairments in models of spinal cord or brain injury [93]. These findings underscore DBS’s potential to restore motor function by modulating neural circuits.

Clinical investigations

A preliminary trial involving two individuals with chronic incomplete SCI revealed that lateral hypothalamic DBS (LH-DBS) immediately enhanced both walking speed and endurance. One participant progressed from using body-weight-supported walking to independent movement, while another regained the ability to climb stairs. Notably, these improvements persisted even after stimulation was discontinued, suggesting the induction of long-term neuroplastic changes [94].

Ongoing clinical trials, including those registered under NCT03053791 and NCT04965727 on ClinicalTrials.gov, continue to assess the efficacy and safety of DBS across various neurological conditions.

Therapies developed and assessed up to the animal testing stage

Cell-Based Therapies

Cell therapies for spinal cord repair have been thoroughly evaluated, including a recent and comprehensive meta-analysis of all clinical studies utilizing cell therapies in SCI [95,96] by Willison et al. [97] NSCs [98,99], pluripotent stem cells [i.e., embryonic stem cells (ESCs) or induced pluripotent stem cells (iPSCs)] [100], mesenchymal stem cells (MSCs) [95,101,102], olfactory sheath cells (OECs) [103], and Schwann cells (SCs) [104] are some of the cells that are most frequently used in cell therapies for SCI.

Most existing cell therapies for SCI use MSCs or other bone marrow-derived stem cells, and there are currently no trials using iPSCs or SCs. Some studies in SCI involve NSCs or OECs. Although numerous investigations on cell therapies in SCI animal models have indicated functional improvement after transplantation, the efficacy of cell therapy in people has not yet been established [97]. Numerous minor clinical investigations failed to demonstrate substantial operational improvement or had design flaws (e.g., absence of proper controls and showing of improved efficacy compared to normal spontaneous recovery) [95]. The use of cell transplantation as an SCI treatment is still in its early stages. There will be interesting new transrational research in the future.

Manipulation of Cell Signaling Pathways

The therapeutic potential of cyclic adenosine monophosphate (cAMP) in SCI: Adenosine is an inhibitory neurotransmitter within the central nervous system (CNS) [105]. Research increasingly highlights that cAMP facilitates nerve regeneration following SCI, offering protective benefits against ischemia, hypoxia, and traumatic brain injuries [106,107]. A study by Qi et al. [108] aimed to evaluate the protective and regenerative effects of cAMP on SCI in rats. A total of 56 male Sprague Dawley rats were divided into three groups: a sham group that underwent surgery without SCI, an SCI group with untreated spinal cord injury, and a cAMP + SCI group that received cAMP treatment via a micropump for three days following SCI. Locomotor function was assessed using BBB scoring, and hind limb strength was evaluated with an inclined plane test. Results showed that cAMP treatment significantly improved movement capacity and hind limb strength compared to the untreated SCI group at multiple time points (days 3, 7, 14, and 21). These findings suggest that cAMP has a protective and therapeutic effect on SCI, highlighting its potential for promoting nerve regeneration and functional recovery.

Phosphatase and tensin homolog (PTEN)/mammalian target of rapamycin (mTOR) regulating pathway: Studies have further revealed that conditional knockout of negative regulators of rapamycin, such as PTEN or tuberous sclerosis complex 1, in adult retinal ganglion cells promotes axon regeneration after optic nerve injury [109, 110]. Modulating intrinsic PTEN/mTOR activity in neurons has been shown to facilitate axon regeneration and functional recovery following SCI in adults [111].

Kar et al. conducted a study demonstrating that microRNA-21 (miR-21) enhances axon growth and protein synthesis by suppressing PTEN, a key inhibitor of the mTOR pathway, which is essential for promoting protein synthesis and axon regeneration [112]. In contrast, microRNA-199a-3p (miR-199a-3p) reduces axon growth and protein synthesis by suppressing mTOR directly, thereby limiting neuronal repair. The injury-induced changes in these miRs specifically the increase in miR-21 and decrease in miR-199a-3p modulate the PTEN/mTOR pathway, influencing the capacity for axon regeneration. These findings highlight miR-21 and miR-199a-3p as potential therapeutic targets for promoting nerve repair and functional recovery following neural injury.

The Glial Scar

The glial scar consists of multiple cell types, including astrocytes, fibroblasts, neural stem/progenitor cells (NSPCs), microglia, macrophages, and immune cells. Its formation is driven by a combination of cellular signals in the surrounding area after an injury and can continue to develop for months following a SCI [113-115]. Injuries to the CNS initiate a glial response that leads to the formation of a glial scar, which serves as a physical and chemical barrier to regeneration. Therefore, reducing glial scar formation or limiting astrocyte aggregation may help remove this obstacle and promote regeneration [116]. Additionally, fibroblasts create an impenetrable barrier to cellular and axonal growth, hindering neural regeneration [117]. The mesenchymal and fibrotic components of the scar can be targeted through genetic interventions or pharmacological treatments to inhibit their proliferation [117]. For instance, administering anti-mitotic medication such as Taxol and epothilone B has been shown to rearrange microtubules, halt cell division, and prevent the migration of fibroblasts to the injury site, thereby enabling axon regeneration and functional recovery [118].

Chondroitin sulfate proteoglycans (CSPGs) released by hyperproliferating, "reactive" astrocytes after CNS damage are considered to be the main candidates for mediating scar-inhibiting activity [119,120]. According to this theory, preclinical studies in rodents have demonstrated that the therapeutic dissolution of a CSPG-rich matrix with chondroitinase ABC benefits axonal regeneration and functional recovery after SCI [120,121]. The specific and highly affine binding receptor for CSPGs is a transmembrane receptor tyrosine phosphatase, or protein tyrosine phosphatases receptor specific for CSPG (PTPσ) [122]. Later, it was shown that LAR, another member of the protein tyrosine phosphatases receptor (PTPR) subfamily, had a strong affinity for CSPGs. Following a lesion of the spine in rodents, intervention with a peptide targeting LAR enhanced axonal regeneration and motor functional recovery [123].

Promoting Axonal Regrowth

Cartilage acidic protein-1B (Crtac1B), also known as lateral olfactory tract usher substance (LOTUS), has recently been identified as a natural inhibitor of the Nogo receptor [124,125]. In vitro studies have demonstrated that LOTUS effectively counteracts axonal growth inhibition mediated by Nogo, MAG, OMgp, chondroitin sulfate proteoglycan, BlyS, and NgR1 signaling, almost entirely neutralizing their effects [126-128].

In a study led by Hirokawa et al., mice with various LOTUS expression levels were used to explore LOTUS's impact on axonal regeneration and recovery after SCI [129]. The study utilized a dorsal over-hemisection model to induce CNS injury, with functional assessments conducted using the Basso Mouse Scale (BMS) [130], footprint analysis, and the grid walking test. Results at 28 days post-injury revealed that mice overexpressing LOTUS (LOTUS-TG) displayed superior motor function compared to wild-type (WT) and LOTUS-deficient (LOTUS-KO) mice, showing higher BMS scores and improved coordination during locomotion. Conversely, LOTUS-KO mice exhibited poorer motor function and limited ankle movements.

Hirokawa and colleagues concluded that LOTUS levels are directly associated with neuronal repair following SCI, highlighting its potential as a therapeutic target to promote axonal regeneration and functional recovery [126]. These findings are consistent with the work of Ito et al. [131], who observed a correlation between LOTUS expression levels and functional recovery in a mouse model of SCI. Additionally, in a mouse model of focal ischemia, LOTUS facilitated axonal sprouting across the midline from the uninjured side to the denervated regions within the medullary reticular formation and cervical spinal cord gray matter [132]. However, it remains unclear whether the reorganized motor pathways influenced by LOTUS are physiologically functional and directly contribute to enhanced functional recovery.

Scaffolding and tissue engineering

Collagen-Based Scaffolds

The study by Cholas et al. investigated the effects of bare collagen and cross-linked collagen implants loaded with laminin and plasmid glial cell line-derived neurotrophic factor (pGDNF) on the repair process in adult rat NSCs over six weeks [133]. They found that collagen implants, either alone or as a scaffold for NSCs or pGDNF, reduced the incidence of cysts in the injury site and promoted axon growth outside the lesion area. Additionally, the scar tissue aligned longitudinally with the spinal cord, indicating collagen's effectiveness as a biomaterial for transporting NSCs and genes and promoting tissue alignment and repair. In another study by Liu et al., electrospun type I collagen nanofibers treated with acetic acid were evaluated for their potential to promote neurite development from dorsal root ganglia (DRG) explants [134]. The aligned collagen fibers encouraged neurite growth and elicited elongated morphology in astrocytes compared to randomly spaced collagen fibers and glass coverslips.

Regarding axonal regeneration in the CNS, collagen-based scaffolds have been shown to inhibit the expression of chondroitin sulfate proteoglycans in scar tissue, stimulate neuronal regeneration, and facilitate the differentiation of endogenous NSCs. This leads to neuronal communication and synaptic connections between injured ends, partially restoring locomotion [135]. Overall, these studies highlight the potential of collagen-based scaffolds in promoting axonal regeneration and tissue repair in SCIs.

*Scaffolds Based on Electrospun Fibers and Films Composed of Poly* *Pro-7β-Estradiol (Pro-E2)*

The study conducted by D'Amato et al. assessed the contact guidance, neurotropism, and neuroprotection of a biomaterial scaffolding made from electrospun fibers and films entirely composed of poly pro-7β-estradiol (pro-e2), releasing estrogens over 1-10 years [136]. They found that neurons grown on electrospun fibers showed significant neurite extension along the fiber direction compared to neurons grown on glass substrates. Additionally, the study examined the capacity of the scaffolding to promote neurite extension and the bioactivity of released E2. The results demonstrated that the continuous release of E2 from the scaffolding significantly enhanced neurite growth compared to a single bolus of E2. These findings highlight the potential of the scaffolding to promote neural growth and suggest its utility in tissue engineering applications.

## Conclusions

The inability of CNS axons to regenerate spontaneously after an injury remains a critical challenge in neuronal regeneration research. Significant progress has been made in understanding the mechanisms that regulate axon growth and the signaling pathways involved. However, major obstacles persist, including the complexity of CNS pathways and the development of therapies that encourage regeneration without causing further damage. Overcoming these challenges is essential for achieving effective neural repair. Despite these hurdles, promising advances offer hope for CNS regeneration. Researchers have identified key molecules and pathways promoting axon growth and are developing innovative treatments such as stem cell therapy, gene therapy, and optogenetics. These approaches, alongside continued scientific exploration, hold the potential to improve recovery and quality of life for individuals with CNS injuries. Sustained efforts in research and collaboration will be critical to achieving meaningful breakthroughs in this field.
